# A user-friendly CRISPR/Cas9 system for mutagenesis of *Neurospora* *crassa*

**DOI:** 10.1038/s41598-024-71540-x

**Published:** 2024-09-03

**Authors:** Stefanie Grüttner, Frank Kempken

**Affiliations:** Abteilung Botanische Genetik und Molekularbiologie, Botanisches Institut und Botanischer Garten, Christian-Albrechts-Universität zu Kiel, Olshausenstraße 40, 24098 Kiel, Germany

**Keywords:** Genetic engineering, Genetic techniques, Neurospora crassa

## Abstract

As a widely used eukaryotic model organism, *Neurospora* *crassa* offers advantages in genetic studies due to its diverse biology and rapid growth. Traditional genetic manipulation methods, such as homologous recombination, require a considerable amount of time and effort. In this study, we present an easy-to-use CRIPSR/Cas9 system for *N.* *crassa*, in which the *cas9* sequence is incorporated into the fungal genome and naked guide RNA is introduced via electroporation. Our approach eliminates the need for constructing multiple vectors, speeding up the mutagenesis process. Using *cyclosporin-resistant-1* (*csr-1*) as a selectable marker gene, we achieved 100% editing efficiency under selection conditions. Furthermore, we successfully edited the non-selectable gene *N-acylethanolamine* *amidohydrolase-2* (*naa-2*), demonstrating the versatility of the system. Combining gRNAs targeting *csr-1* and *naa-2* simultaneously increased the probability of finding mutants carrying the non-selectable mutation. The system is not only user-friendly but also effective, providing a rapid and efficient method for generating loss-of-function mutants in *N.* *crassa* compared to traditional methods.

## Introduction

Due to its diverse biology, ease of cultivation, and rapid growth rate, *Neurospora* *crassa* is widely used as an eukaryotic model organism*.* As a result of its facile genetics, it is often used in genetic studies^1^. To characterize or modify the functions of genes, manipulation on the genome level is the most commonly used strategy. Therefore, tools for manipulating the fungal genome are indispensable. A more traditional way to manipulate a genome is to use mutagens like UV-irradiation or chemical agents that lead to changes in the DNA. This method is rather random and usually creates more than just one mutation in the genome. Finding these mutations is tedious and narrowing down the resulting phenotype to one specific mutation is difficult. Targeted mutagenesis is a better choice for analyzing the function of a specific gene. In *N.* *crassa,* homologous recombination is the standard method for creating knockout mutants or overexpression lines. This method requires constructing a vector containing homologous flanking sequences together with a gene of interest and/or a selection marker^2^. The vector needs to be transformed into a suitable *N.* *crassa* strain, such as a mutant strain that lacks the non-homologous end joining (NHEJ) repair mechanism^3,4^. After obtaining homokaryotic transformants through a cross, microconidia passage or serial transfer of macroconidia, the homokaryotic insertion is verified by Southern-blot or PCR^4,5^. The entire process takes approximately 7 weeks to complete^6^, which is^7^time-consuming, labor-intensive, inefficient (when not using the NHEJ deficient strain), and limited due to the availability of selection markers^8^.To overcome these problems, CRISPR/Cas9 (short for Clustered Regularly Interspaced Short Palindromic Repeats/CRISPR associated proteins) offers an attractive alternative.

Originally, the CRISPR/Cas9 system is a part of the bacterial adaptive immune system against phages and foreign DNA and was adapted as a genome editing tool^9^. Its application allows for precise and efficient genome editing, by creating insertions or deletions (indels) of a few base pairs or even the integration of heterologous or modified endogenous genes^10^. The system consists of two main components. The Cas9 endonuclease and a guide RNA (gRNA). The gRNA is an RNA-duplex of the crRNA (CRISPR associated RNA) needed for recognition of the target sequence and the tracrRNA (trans activating crRNA) required for the interaction with and the activation of Cas9^11^. The gRNA and the Cas9 nuclease form a complex that binds randomly to the DNA. An RNA/DNA heteroduplex is formed once the respective recognition site followed by the protospacer adjacent motif (PAM) 5′-NGG-3′ (in case of *S.* *pyogenes* derived Cas9) is detected^10^. To form the heteroduplex, the first eight nucleotides following the PAM must be complementary to the gRNA. The Cas9 endonuclease then creates a DNA double-strand break (DSB), which will be repaired by the cell’s repair mechanisms: (1) non-homologous end joining (NHEJ) can lead to indels at the site of the DSB or (2) homologous recombination-directed repair (HDR) may allow the insertion of donor DNA flanked by homologous sequences^11^.

In 2013, the first fungal CRISPR/Cas9 system was developed for yeast^12^, followed by the filamentous fungi *Trichoderma* *reseei* and several *Aspergilli* in 2015^13,14^. Several other fungal CRISPR-Cas systems followed, including *N.* *crassa*^15,16^. However, the published *N.* *crassa* system requires the transformation of separate plasmids simultaneously, carrying the *cas9*, gRNA and donor DNA sequences. Consequently, this system did not become a standard tool for *N.* *crassa*. Here we describe a novel CRISPR/Cas9 system for easier handling, where the *cas9* sequence is integrated into the fungi’s genome and the gRNA is transformed as naked RNA, making it unnecessary to clone and transform a *cas9/*gRNA carrying plasmid.

## Results

### Design of the CRISPR/Cas9 system for *Neurospora crassa*

**Fig. 1:**
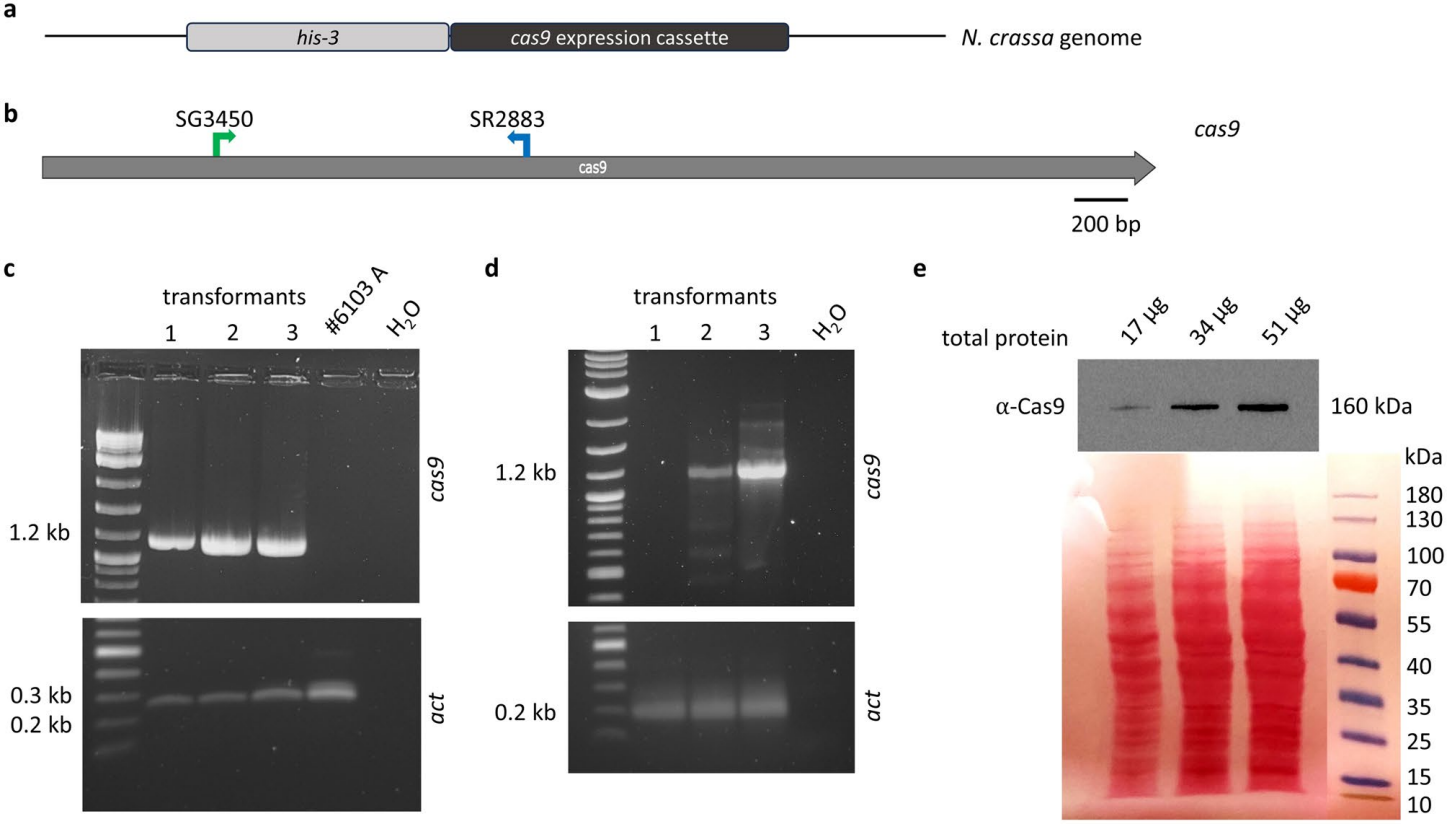
Schematic of *cas9* expression cassette integrated into the genome and verification of its integration, transcription and expression. (**a**) Illustration (not to scale) of the position of the *cas9* expression cassette in the genome of *N.* *crassa* strain #6103. (**b**) Illustration of the *cas9* gene with oligonucleotides used for amplifying part of the *cas9* sequence from genomic DNA and total RNA. Green arrow: forward oligonucleotide (SG3450); blue arrow: revers oligonucleotide (SR2883), (**c**) PCR amplifying part of the *cas9* sequence and *actin* (as a control) from genomic DNA of the transformed #6103 strain (1–3). Genomic DNA of #6103 before transformation and water were used as negative controls. Verifying its integration into the *N.* *crassa* genome. Gel image has been cropped, for full size gel image see Supplementary Fig. 1. (**d**) RT-PCR amplifying part of the cDNA of the *cas9* and *actin* (as a control) transcripts, using RNA of the transformants that were tested positive for the *cas9* DNA sequence (1–3) and water as a negative control. For verification of the *cas9* transcription. Gel image has been cropped, for full size gel image see Supplementary Fig. 1. (**e**) Immunoblot analysis of Cas9 expression of the Cas9-strain NcCas9SG using a Cas9 antibody. 17, 34, and 51 µg of total proteins were used in each lane, as indicated. The molecular weight of the respective protein is given in kDa. For loading control the ponceau-red stained membrane is shown. Image has been cropped, for full size membrane image see Supplementary Fig. 2.

In the presented work we use a step-wise introduction of Cas9 and the gRNA into the fungus. The *cas9* sequence, optimized according to human codon usage, was amplified from the pSpCas9n(BB)-2A-GFP (PX461) vector containing the nucleoplasmin nuclear localization signal (NLS) at its 3′ end. This sequence was cloned into a fungal transformation vector, which was integrated at the *his-3*-locus of the *N.* *crassa* strain #6103 (see Fig. 1a). The expression of *cas9* is under the control of the *ccg1*-promotor of *N.* *crassa*. The resulting strain was named NcCas9SG.

**Fig. 2:**
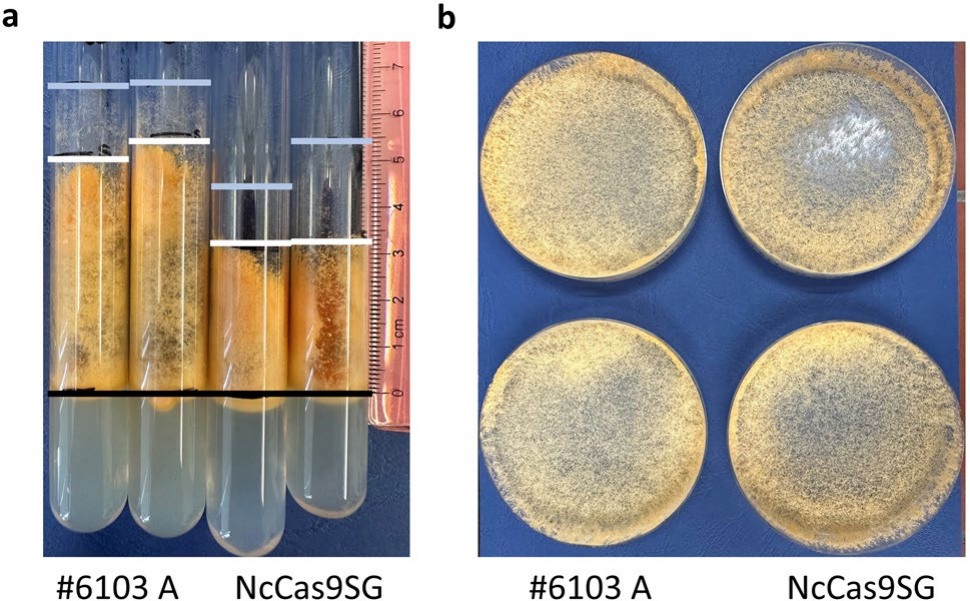
Phenotypical comparison of NcCas9SG and #6103 (**a**) Comparison of aerial hyphae formation of NcCas9SG and #6103. VMM + S + his slant tubes were inoculated with 10^6^ spores of the respective strain and after seven days at 25 °C growth was measured. The black line marks the starting point of the measurement. It was distinguished between the height up to where spore formation took place (white line) and the height of the growing hyphae (light blue). (**b**) Comparison of lateral hyphae growth of NcCas9SG and #6103. VMM + S + his plates were inoculated with 10^6^ spores of the respective strain. After three days at 25 °C growth was compared.

The successful integration of the *cas9* sequence was verified by PCR using oligonucleotides SG3450 and SR2883 for three transformants (see Fig. 1b and c) as well as by Southern blot (see Supplementary Fig. S3). Expression on the transcript level was confirmed for transformants number two and three, both stemming from a microconidia passage of the same independent transformant, by RT-PCR (see Fig. 1d). Transformant number three was chosen for all following experiments. To analyze the expression of Cas9 on the protein level a Western blot using an anti-Cas9 antibody was done, confirming that the Cas9 protein is synthesized in the transformant (see Fig. 1e). To check for undesired adverse effects on growth behavior or conidiation we phenotypically compared NcCas9SG to the host strain #6103 (see Fig. 2a and b). Aerial hyphae growth of NcCas9SG was lower compared to #6103. When grown for 7 days at 25 °C in a glass tube aerial hyphae of strain #6103 grew up to 5.7 cm ± 0.29, carrying spores up to a height of 4.57 cm ± 0.21 in the tube, while the aerial hyphae of NcCas9SG only reached a height of 3.95 cm ± 0.71, carrying spores up to a height of 2.67 cm ± 0.19. The lateral growth behavior on a petri dish containing VMM + S agar on the other hand did not show any significant differences. Also, the number of produced spores did not differ significantly with 5.59 × 10^8^ spores/ml ± 0.55 × 10^8^ for #6103 and 5.38 × 10^8^ spores/ml ± 0.99 × 10^8^ for NcCas9SG. Therefore, we conclude, that the expression of Cas9 does not significantly limit the growth of NcCas9SG, thus making it useful for a mutagenesis system.

After confirming Cas9 expression in NcCas9SG, macroconidia were isolated from the strain and used for the transformation of gRNA. For the introduction of the gRNA into the cell, synthetic crRNA/tracrRNA duplexes instead of plasmid DNA were transformed into macroconidia via electroporation. Thereby avoiding the problems that can occur when the gRNA has to be transcribed from the DNA template^17^.

### Editing of the *csr-1* gene with CRISPR/Cas9

**Fig. 3:**
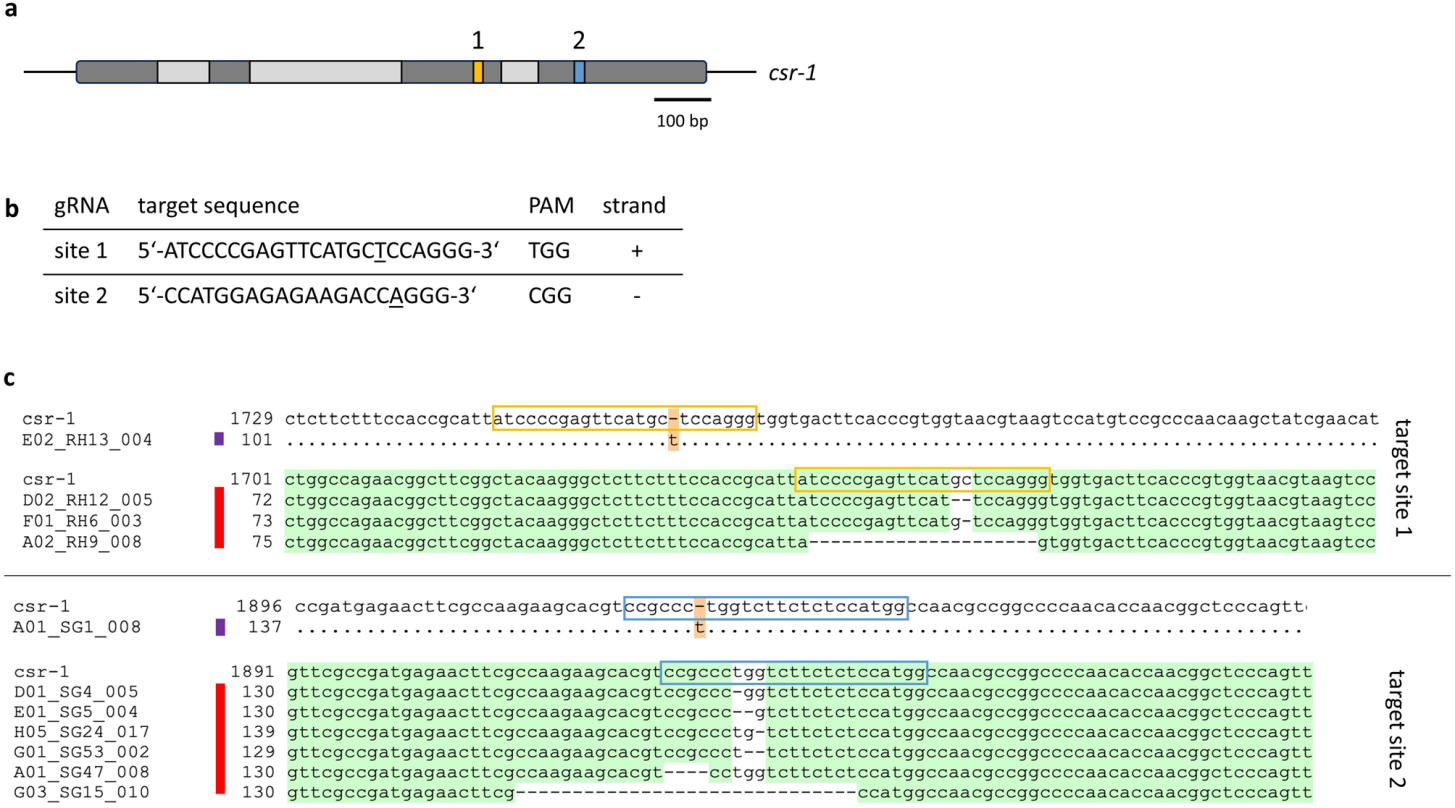
Editing of the *csr-1* gene. (**a**) The *csr-1* gene with exons (dark grey) and introns (light grey) is shown with the gRNA binding positions indicated in yellow (gRNA-c1) and blue (gRNA-c2); introns in light grey, exons in dark grey. (**b**) gRNA sequences. (**c**) Examples of sequence alignments of *csr-1* sequences from edited fungi with the wild type *csr-1* sequence. Deletions are marked with a red and insertions with a purple line. Dots and green background indicate identical nucleotides, dashes indicate missing nucleotides, orange background indicates additional nucleotides.

To test our CRISPR/Cas9 system for its functionality, a selectable marker gene was chosen as a target for editing. The *cyclosporin* *resistant-1* (*csr-1*) gene (NCU00726) codes for a peptidyl-prolyl cis–trans isomerase. Mutations in the *csr-1* gene lead to resistance against cyclosporin A (CsA)^18^, allowing for an easy identification of putatively mutated *N.* *crassa* colonies on CsA containing medium. Two gRNAs, designated gRNA-c1 and gRNA-c2 (see Fig. 3a and b), targeting different regions of the *csr-1* sequence were used. gRNA-c1 targets position 126–148 of exon 3 and gRNA-c2 targets position 66–88 of exon 4.

When plating transformed macroconidia on VMM + SGF without CsA, an editing efficiency of 7.35% ± 1.38 for gRNA-c1 and 11.89% ± 4.78 for gRNA-c2 was determined. When plating transformed macroconidia on selection media (VMM + SGF + CsA) 100% of the colonies obtained (8/8 colonies, using gRNA-c1 and 24/24 colonies, using gRNA-c2) showed a mutation at the predicted site. 1, 2 or 3 µl of 100 µM gRNA-c2 were used to transform macroconidia. No significant difference in the number of obtained colonies was observed on selection media (see Supplementary Fig. S4). Using 1 µl of gRNA resulted in 223 colonies/plate ± 24, 2 µl resulted in 246.5 colonies/plate ± 2.1, and 3 µl resulted in 209 colonies/plate ± 4.2. Hence, 2 µl of 100 µM of gRNA was considered to saturate the system. The mutations at the predicted site were determined by amplification of the target region via PCR and sequencing. Figure 3c shows examples of different types of mutations at the predicted sites for the two gRNAs. All observed types of mutations are summarized in Table 1. These findings are direct proof of the suitability of our newly designed *N.* *crassa* CRISPR/Cas9 system.

**Table 1:** Different types of mutations in the *csr-1* gene created by the CRISPR/Cas9 system.

	Type of mutation	Abundancy
Target site 1	1 bp insertion	8/16
	1 bp deletion	2/16
	2 bp deletion	3/16
	Deletion > 2 bp	3/16
Target site 2	1 bp insertion	16/53
	2 bp insertion	4/53
	Insertion > 2 bp	1/53
	1 bp deletion	12/53
	2 bp deletion	11/53
	Deletion > 2 bp	9/53

### Editing of a non-selectable gene with CRIPSR/Cas9

**Fig. 4:**
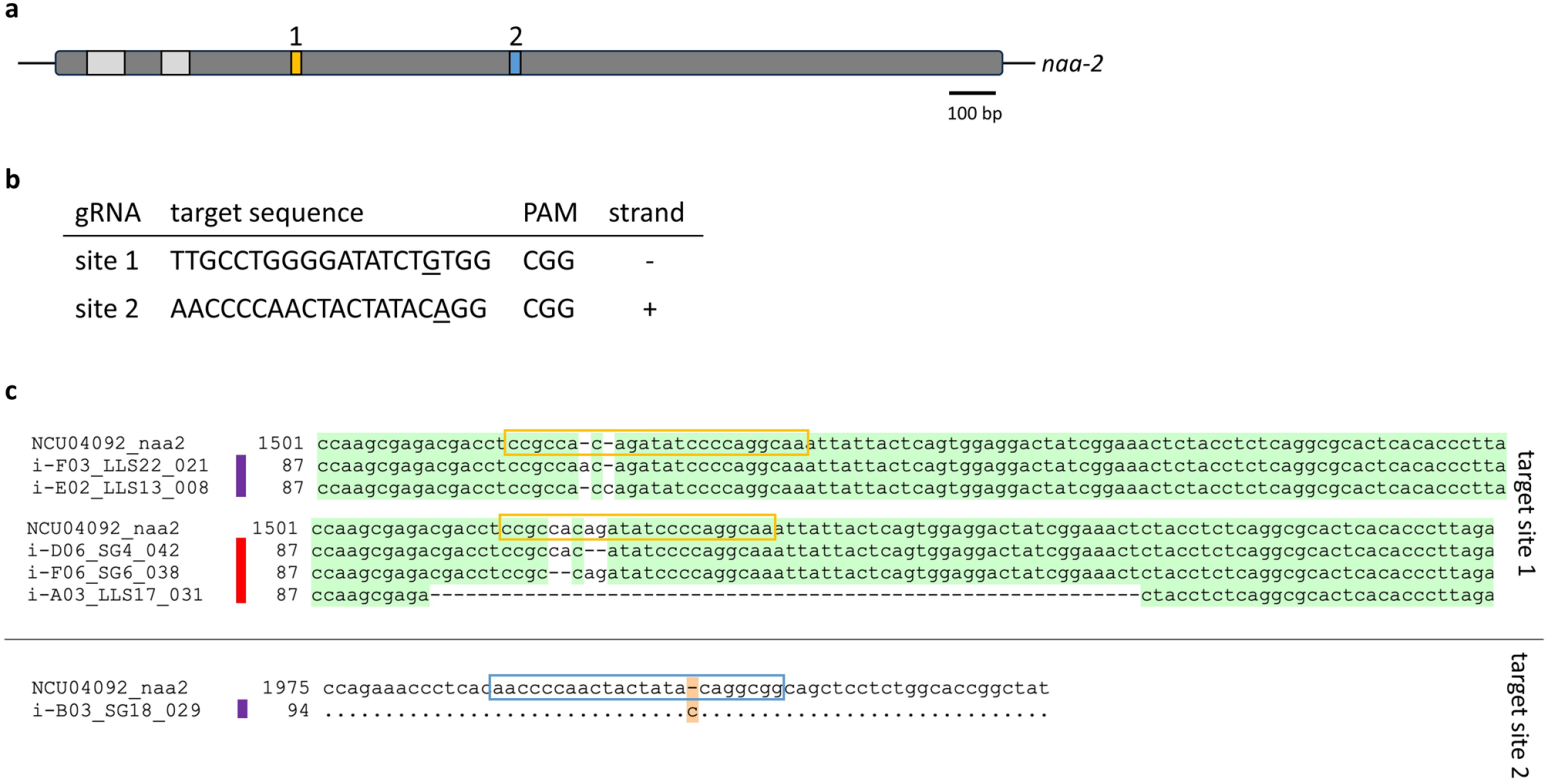
Editing of the *naa-2* gene. (**a**) The *naa-2* gene with exons (dark grey) and introns (light grey) is shown with the gRNA binding positions indicated in yellow (gRNA-n1) and blue (gRNA-n2). (**b**) gRNA sequences. (**c**) Examples of sequence alignments of *naa-2* sequences from edited fungi with the wild type *naa-2* sequence. Deletions are marked with a red and insertions with a purple line. Dots and green background indicate identical nucleotides, dashes indicate missing nucleotides, orange background indicates additional nucleotides.

Next, a non-selectable gene encoding N-acylethanolamine amidohydrolase-2 (*naa-2*; NCU04092) involved in the auxin biosynthesis pathway in *N.* *crassa*^19^, was targeted. Again, two different gRNAs (see Fig. 4a and b) were designed: gRNA-n1 (targeting sequence position 211–233 of exon 3) and gRNA-n2 (targeting sequence position 683–705 of exon 3). After the transformation of NcCas9SG macroconidia with the respective gRNA, the conidia were transferred on VMM + SGF plates and 19 randomly picked colonies for each gRNA were screened for a putative mutation. In case of gRNA-n1 5.26% (1/19 colonies) of the analyzed colonies carried a mutation at the predicted site while no mutations (0/19 colonies) were found for gRNA-n2. These findings show, that mutations may be found even without a selective marker, albeit it is laborious and time consuming.

### Editing of more than one gene at a time using CRIPSR/Cas9

Typically, the CRISPR/Cas9 system allows for editing of multiple genes at the same time by introducing several gRNAs to a cell that target different sequences at once. This technique was shown to work in several filamentous fungi such as *Aspergillus* *fumigatus*^20^, *A.* *niger*^21^ and *Beauveria* *bassiana*^22^*.* We set out to use this technique in *N.* *crassa*, using a combination of two gRNAs at the same time (gRNA-c2/gRNA-n1 and gRNA-c2/gRNA-n2) to transform NcCas9SG macroconidia. These gRNA combinations seemed promising to minimize the expenditure of time when screening for the mutation since one gRNA targeted the *csr-1* gene, allowing the selection for a CRISPR event via CsA. The combination of gRNA-c2 with either gRNA-n1 or gRNA-n2 increased the efficiency of observing *naa-2* mutations significantly (summarized in Table 2). In case of gRNA-n1 55.5% (11/18) of the colonies carried a mutation at the predicted site. This is a tenfold increase compared to the previous transformation without the gRNA-c2 where only 5.26% of the analyzed colonies carried a mutation. The same increase was observed for gRNA-n2 where 16.7% (1/6) colonies carried a mutation at the predicted site. Figure 4c shows examples of different types of mutations at the predicted sites in the *naa-2* sequence for both gRNAs. All observed types of mutations are summarized in Table 3. Analysis of the target site for gRNA-c2 showed again, that 100% of the colonies carried the mutation at the predicted site, meaning that 50% (12/24) of the analyzed colonies carried a mutation at both targeted genes. Therefore, by using this approach, it becomes much easier to detect the individuals carrying the desired mutation.

**Table 2:** Percentage of analyzed colonies carrying a mutation in the *naa-2* gene after editing using gRNA-n1/gRNA-n2 alone or in combination with gRNA-c2.

gRNA	Alone (%)	In combination with gRNA-c2 (%)
gRNA-n1	5.26	55.5
gRNA-n2	0	16.7

**Table 3:** Different types of mutations in the *naa-2* gene created by the CRISPR/Cas9 system.

	Type of mutation	Abundancy
Target site 1	1 bp insertion	4/12
	2 bp insertion	1/12
	2 bp deletion	4/12
	Deletion > 2 bp	3/12
Target site 2	1 bp insertion	1/1

## Discussion

We developed a simple and versatile CRISPR/Cas9 system that can be used efficiently in *Neurospora* *crassa*. The most common method used in other filamentous fungi, such as *Aspergillus* or *Penicillium* species, is to introduce the *cas9* and gRNA sequences to the cell through self-replicating plasmids^14,23^. Similarly, non-replicating plasmids were used to introduce both sequences to the cell in the already established system for *N.* *crassa*^16^. We aimed to avoid constructing multiple vectors which both mentioned methods require, as it is a time-consuming process. In our system, the Cas9 sequence was integrated into the fungal genome. Then, the gRNA was introduced into the cell as an RNA duplex consisting of crRNA and tracrRNA using electroporation. This method is similar to the procedure used in *Fusarium* *fujikuroi* and *Trichoderma* *reesei*^13,24^. Hence, using the NcCas9SG strain eliminates the need for any vectors, greatly speeding up the editing of single or multiple genes at a time. Introducing the gRNA to the cell as an RNA duplex avoids problems with gRNA gene transcription from vector DNA^25^. Furthermore, this can greatly increase the efficiency compared to the systems^12^ that require the transformation of two separate vectors for Cas9 and the gRNA, respectively. Mainly due to the inefficacy of combining two separate vectors for gRNA and Cas9^20^, for example in *Aspergillus* *fumigatus,* it is more effective to use a single vector for both^20^. However, a single vector coding for Cas9 and the gRNA, still requires creating a new vector for each mutagenesis experiment. There is also a potential risk of random genome integration of the plasmid^26^ or small DNA fragments resulting from intracellular degradation of the plasmids^27^ when transforming plasmids. However, this risk can be avoided by using our system, as there is no need to transform any additional vector. An alternative approach would have been to transform the Cas9-gRNA complex as a ribonucleoprotein, which has been successfully achieved in *Fusarium* *oxysporum* and *Aspergillus* *fumigatus*^28,29^. However, as we aimed to develop an easy-to-use version of the CRISPR/Cas9 system, we did not consider this method since delivering ribonucleoproteins to cells is challenging and rarely employed^25^.

Another concern involves off-target mutations that the CRISPR/Cas9 system may potentially cause^30^. However, studies have shown that limiting the presence of the Cas9-gRNA complex can help to reduce these off-target effects^27^. An inducible Cas9 would be an obvious alternative but has yet to be established. Therefore, in our CRISPR/Cas9 system, the presence of Cas9 in the cells is not restricted, but the presence of the Cas9-gRNA complex is limited. This limitation alone can help minimize the off-target effects of the system. For example, in *Aspergillus* *niger*, it was demonstrated that when Cas9 is used in combination with a gRNA, there is an increase in random mutations, whereas Cas9 alone does not lead to this outcome^31^. It has also been indicated that most of the observed off-target mutations are more likely due to the transformation process itself rather than the CRISPR/Cas9 system, as observed in *A.* *niger*, *A.* *fumigatus* and *Ustilago* *maydis*^31–33^. To ensure the elimination of off-target effects occurring after performing the desired mutagenesis, it is possible to out-cross the *cas9* sequence.

Aside from being user-friendly, our system is also effective. Editing the selectable *csr-1* gene resulted in an increased editing efficiency from 7.35% (gRNA-c1)/11.89% (gRNA-c2) to 100% when selection pressure was applied. Combining the editing of the non-selectable gene with the editing of the selectable *csr-1* gene resulted in a tenfold increase in finding mutations of the non-selectable genes. Most likely, the increase in finding mutations while using this combination of gRNAs (gRNA-c2/-n1 or gRNA-c2/-n2) is due to the ability to select for an editing event using CsA selection plates, rather than the potential for the two gRNAs to act as carriers for each other. This is supported by the fact that the combination of gRNA-n1 and -n2 did not increase the desired mutations. In previous studies^18^ and this study, no obvious uncharacteristic phenotype for the *csr-1* mutants was observed. The *csr-1* locus is frequently used in *N.* *crassa* as a marker for gene integration through homologous recombination. For example in studies on RIP and meiotic silencing^34,35^, indicating that knocking out *csr-1* does not affect the fungus’ asexual or sexual development. This suggests, that *csr-1* is a suitable marker for the CRISPR/Cas9 system presented here. As *N.* *crassa* macroconidia are multi-nuclear and *csr-1* mutations tend to result in homokaryons^18^ using *csr-1* as a marker offers an advantage. Obtaining homokaryotic strains after transformation usually involves a cross, microconidia passage, or a serial transfer of macroconidia^5^ which can be a laborious task. However, this becomes unnecessary when utilizing the CRISPR/Cas9 system in combination with *csr-1* as a marker.

**Fig. 5:**
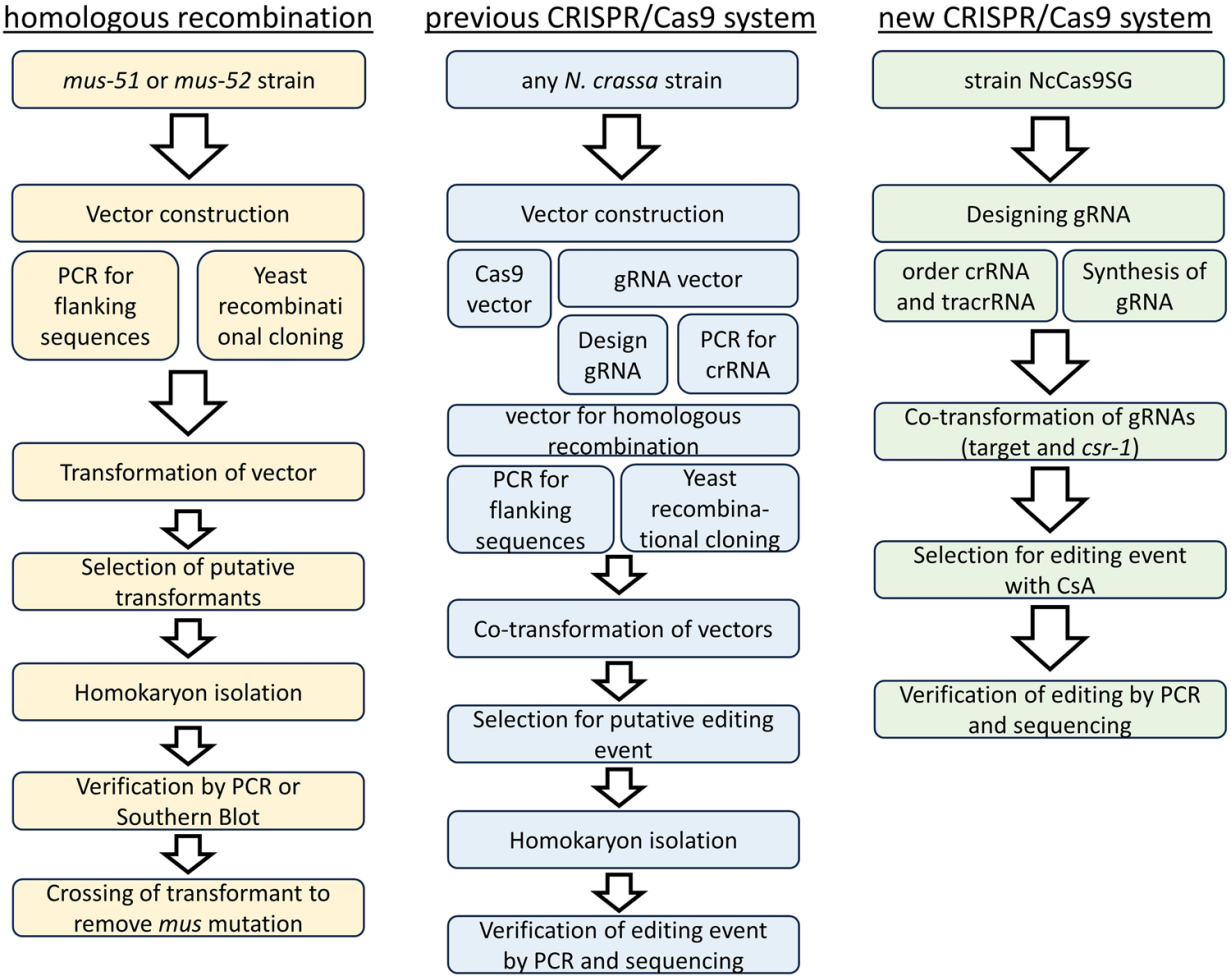
Comparison of different mutagenesis methods for *N.* *crassa.*

In conclusion, we present a fast and easy-to-handle CRISPR/Cas9 system for creating loss-of-function mutants of *N.* *crassa*. The editing efficiency of our system ranges from 7.35 to 11.89% depending on the used gRNA, comparable to the efficiency of homologous recombination in a wild-type background, which is less than 10%^3,4^ (see Table 4). Although this efficiency is not higher compared to the homologous recombination, it can be improved by using a selective marker, such as the *csr-1* gene. It is common to use a selection marker when performing mutagenesis in *N.* *crassa*, and in this case, it significantly increases the efficiency. If the *csr-1* mutation is not desired for further analyses of the mutated strain it can be out-crossed after the mutagenesis, eliminating any negative effects that may occur due to the *csr-1* mutation. This makes it a good alternative when not using the *mus* strains, especially when editing of more than one gene at a time is needed. Moreover, Fig. 5 presents an overview of the various mutagenesis systems developed for *Neurospora* *crassa*, highlighting that our system requires fewer steps and therefore less time to achieve targeted mutagenesis compared to the other systems. As a result, this system is a very user-friendly and time-saving method for creating loss-of-function mutants in *N.* *crassa* which will promote research.

**Table 4:** Comparison of the efficiency of different mutagenesis systems in *Neurospora* *crassa.*

System	Efficiency	References
Homologous recombination in *mus* background	98.9%	^4^
Homologous recombination in wild type	< 10%	^3,4^
Previously described CRISPR/Cas9 system	not given	^16^
CRISPR/Cas9 system in this work	7.35–11.89%	This work

## Methods

### Strains

The histidine auxotrophic *N.* *crassa* strain FGSC #6103 (his-3 (Y234M723) mat A) provided by the Fungal Genetics Stock Center (FGSC; Kansas City, MO, USA) was used for transformation with vector pSG897.

The bacterial strain *E.* *coli* XL1-Blue (*recA1*, *endA1*, *gyrA96*, *thi-1*, *hsdR17*, *supE44*, *relA1*, and *lac* *F’proAB* *lacIqZΔM15* *Tn10* *(Tetr)*) (200249, Stratagene) was used for the propagation of vector constructs.

### Media and growth conditions

For the cultivation of *N.* *crassa,* Vogel’s minimal medium^36^ with 2% sucrose (VMM + S) (supplemented with 0.02% histidine in case of the *his*-3 *N.* *crassa* strain FGSC #6103) was used and for maintaining single colonies on plates Vogel’s minimal medium with 1% sorbose, 0.05% glucose, and 0.05% fructose (VMM + SGF) was used. *N.* *crassa* microconidia production was induced on a synthetic cross medium (SC) supplemented with 0.06% iodoacetate as described in^37^. Fungi were cultivated in a climate chamber at 25 °C under long-day conditions.

The *E.* *coli* strains were cultivated in a Luria Broth culture medium (1% (w/v) tryptone, 0.5% (w/v) yeast extract, 0.5% (w/v) NaCl, pH 7.5) supplemented with the required antibiotics at 37 °C and 250 rpm.

### Generation of *N. crassa* Cas9 strain NcCas9SG

The strain NcCas9SG was created by transforming *N.* *crassa* strain FGSC #6103 (his-3 (Y234M723) mat A) with the vector construct pSG897 carrying the *Spcas9* sequence with a 3′ nucleoplasmin NLS. The *Spcas9* sequence was amplified from the plasmid pSPCas9(BB)-2A-GFP (Addgene plasmid ID: 48138) using the oligonucleotides SG3449 and SG3444 adding an *AscI* and *SwaI* recognition site to the PCR product. Blunt-end PCR products were subcloned using a CloneJet PCR cloning Kit (Thermo Fisher Scientific, Waltham, MA, USA) according to the manufacturer’s recommendation. After hydrolysis of the subcloned product and the vector pMM536^38^ with the corresponding restriction endonucleases from NEB (Ipswich, MA, USA), according to the standard protocol, gel elution was performed with NucleoSpin Gel and PCR Clean-Up Kit (Macherey–Nagel), according to the manufacturer’s instructions. Followed by ligation with T4 DNA-ligase by NEB (Ipswich, MA, USA) according to the standard protocol, creating vector pSG897. The transformation process was carried out as described in^39^ and the transformants were selected using VMM + SGF plates without histidine.

### gRNA design and synthesis

The gRNAs were designed using the CHOPCHOP v3 website (https://chopchop.cbu.uib.no)^40^. For gRNA synthesis two RNA oligonucleotides (crRNA and tracrRNA) synthesized by IDT (integrated DNA technologies, Coralville, Iowa, USA) were ordered (https://eu.idtdna.com/pages). For synthesis of the gRNA equimolar amounts of crRNA and tracrRNA were combined in the IDT-Duplex buffer followed by a 5 min incubation at 95 °C, yielding 100 µM gRNA. After cooling the formed gRNA to room temperature, it can be used for electroporation or stored at − 20 °C.

### Transformation of gRNA

The *N.* *crassa* transformation method for plasmids as described in^39^ was adapted to transform macroconidia of NcCas9SG with the synthesized gRNA(s). 40 µl of macroconidia (2.5 × 10^9^ conidia/ml) were mixed with 2 µl of 100 µM gRNA and kept on ice for 5 min. After transferring the mixture to a cuvette, electroporation was performed using the following parameter: 25 µF, 600 Ω and 1.5 kV. After the pulse the macroconidia were mixed with 1 ml of 1 M sorbitol and incubated at room temperature for 10 min. Without selection pressure the mixture was diluted 1:10,000 or 1:1000 and 100 µl of the dilution were mixed with 8 ml VMM + SGF top-agar and plated on VMM + SGF plates. With selection pressure undiluted 200 µl of the mixture were mixed with top agar. When targeting the *csr-1* gene 5 µg/ml cyclosporine-A was added to the media for selection. Plates were incubated in a climate chamber at 25 °C under long-day conditions for 5–7 days and grown colonies were further analyzed for mutations at the target sites.

### DNA and RNA isolation

DNA isolation was performed either from 3 days old mycelium grown at 25 °C in liquid VMM + S by using the Quick-DNA Fungal/Bacterial Miniprep Kit (Zymo Research Europe, Freiburg, Germany) according to the manufacturer’s instructions or by lysing macroconidia in 50 µl TE buffer for 10 min at 99 °C, followed by a 10 min incubation on ice and a centrifugation step (15,000 × g for 5 min). 40 µl of the supernatant were then transferred to a clean tube and used as PCR template.

Bacterial plasmid DNA was isolated using the NucleoSpin Plasmid Easypure Kit, according to the manufacturer’s instructions (Macherey–Nagel, Düren, Germany).

RNA isolation from 3–4 day old mycelium was performed with peqGold TriFAST (Peqlab, Erlangen, Germany), according to the manufacturer’s instructions, followed by a DNase treatment step using DNase I (ThermoFisher Scientific) according to the standard protocol.

### PCR, RT-PCR and sanger sequencing

PCR was performed as previously described in^38^ using the Taq polymerase from NEB (Ipswich, MA, USA).

For analyzing transcripts, RT-PCR was performed using the OneTaq One-Step RT-PCR Kit from NEB (Ipswich, MA, USA) according to the manufacturer’s instructions.

Sanger sequencing was performed by the Institute of Clinical Molecular Biology (IKMB) in Kiel.

The oligonucleotides used in our study were synthesized by Eurofins MWG Operon (Ebersberg, Germany) and are listed in Table 5.

**Table 5:** Oligonucleotides used in this work.

Oligonucleotide	Sequence 5′-3′	Purpose
SG3449	GGCGCGCCATGGACAAGAAGTACAGCATCGGCCTGGAC	Amplification of *cas9* sequence for cloning; forward
SG3444	ATTTAAATTTACTTTTTCTTTTTTGCCTGGCCGGCCTTTTTC	Amplification of *cas9* sequence for cloning; revers
SG3450	TCTGCCAGACTGAGCAAGAG	Test for *cas9* integration into the *N.* *crassa* genome,RT-PCR and probe amplification; forward
SR2883	TCAGCAGATCGTGGTATGTG	Test for *cas9* integration into the *N.* *crassa* genome,RT-PCR and probe amplification; revers
SG3500	ATCGGATGTCTAACTCCCAATG	Amplification of *csr-1* target site 1; forward
SG3501	AGTGCCCTGTTCAGAGAGGTTA	Amplification of *csr-1* target site 1; forward
SG3496	CTCTTCTTTCCACCGCATTATC	Amplification of *csr-1* target site 2; forward
SG3497	ACAGTGGTGACGAAGAACTGG	Amplification of *csr-1* target site 2; revers
SG3511	TCAGATCGTACCCTTTCCAGAT	Amplification of *naa-2* target site 1 and 2; forward; Sequencing of *naa-2* target site 1
SG3510	AAGACCGATAGGGATGAGACC	Amplification of *naa-2* target site 1 and 2; revers
SG3509	CCTTGGCTCGAAAAACATCTAC	Sequencing of *naa-2* target site 2
SG3702	CTTGCTCATGTGCTCAAGAC	Amplification of *his3* probe; forward
SG3703	CGTCCGATGCCATCTACAAG	Amplification of *his3* probe; revers

### Southern blot

*N.* *crassa* was cultivated in 50 ml VMM + S liquid medium for 3 days at 25 °C (80 rpm), for isolating DNA. After filtering with sterile mull and washing the mycelium with water it was ground in liquid nitrogen. The powder was resuspended in 50% (v/v) DNA lysis buffer (10 mM Tris–HCl pH 8.0; 1 mM EDTA; 100 mM NaCl; 2% (v/v) SDS) followed by phenol extraction with 1 vol phenol. The aqueous phase was extracted twice with 1 vol phenol–chloroform–isoamyl alcohol (25:24:1) and 100 µg of RNase A was added to the aqueous phase, followed by additional phenol extraction and ethanol precipitation. The resulting DNA pellets were resuspended in TE buffer (20 mM Tris–HCl pH 7.5, 0.1 mM EDTA). The isolated DNA was used for southern blotting as previously described^41^. The probes were digoxigenin-labeled generated with the PCR digoxigenin labeling Mix (Roche, Mannheim, Germany), following the manufacturer’s protocol. Oligonucleotides used for the probes are found in Table 5.

### Protein analysis

For isolating proteins, *N.* *crassa* was cultivated in 50 ml VMM + S liquid medium for 3 days at 25 °C (80 rpm). After filtering with sterile mull and washing the mycelium with water it was ground in liquid nitrogen. The powder was resuspended in 50% (v/v) protein extraction buffer (20 mM HEPES–KOH pH 7.9; 100 mM KCl; 20% (v/v) glycerol; 0.2 mM EDTA and 10 µl/ml buffer of protease inhibitor cocktail (P9599) from SIGMA-Aldrich (St. Louis, MO, USA)) and incubated on ice for 10 min. To remove the cell debris, the samples were centrifuged at 12,000 × g for 20 min (4 °C). The supernatant was used for SDS-PAGE and Western-Blot.

Total proteins were separated on 10% SDS–PAGE gels (TGX stain-free FastCast Acrylamide Kit 10%; Bio-Rad, Hercules, CA, USA) and electro transferred to PVDF membrane using the Trans-Blot Turbo™ Transfer System according to the manufacturer’s recommendations (Bio-Rad, Hercules, CA, USA). Protein immunodetection was performed according to the standard procedure using the primary antibody Cas9 (7A9-3A3) Mouse mAb (1:3000 dilution; Cell Signaling, Danvers, MA, USA) and a secondary anti-mouse IgG horseradish peroxidase-conjugated antibody (1:5000 dilution, Cell Signaling, Danvers, MA, USA). The membrane was cut before antibody detection. Signals were detected by chemiluminescence using the GelDoc Go gel imaging system (BioRad, Hercules, CA, USA).

## Supplementary Information

Supplementary Figures.

## Data Availability

The datasets generated and/or analysed during the current study are available in the opendata@uni-kiel the research data repository of the Christian-Albrechts-University Kiel. It can be accessed via the DOI: 10.57892/100-39101.57892/100-39 or the following link: https://opendata.uni-kiel.de/receive/fdr_mods_00000039.
