# Supplementary material for: A user-friendly CRISPR/Cas9 system for mutagenesis of Neurospora crassa
Source: Sci Rep. 2024 Sep 3;14:20469. doi: 10.1038/s41598-024-71540-x (PMC11372047; doi:10.1038/s41598-024-71540-x)
Supplement: Supplementary file 1 — Supplementary Figures. [file 41598_2024_71540_MOESM1_ESM.pdf]

Supplementary Information

A user-friendly CRISPR/Cas9 system for mutagenesis of *Neurospora crassa*

Stefanie Grüttner[1,*], Frank Kempken[1]

[1]Abteilung Botanische Genetik und Molekularbiologie, Botanisches Institut und Botanischer Garten, Christian-Albrechts-Universität zu Kiel, Olshausenstraße 40, 24098 Kiel, Germany

[*]Correspondence: Stefanie Grüttner (sgruettner@bot.uni-kiel.de)

Supplementary Figure 1. Full-length gels of DNA-gels shown in Figure 1c and d

Supplementary Figure 2. Full-length blot of western blot shown in Figure 1e

Supplementary Figure 3. Southern blot to confirm the *cas9* integration into the genome

Supplementary Figure 4. N. crassa colonies on CsA containing VMM+SGF plates after transformation with different gRNA-c2 amounts

a

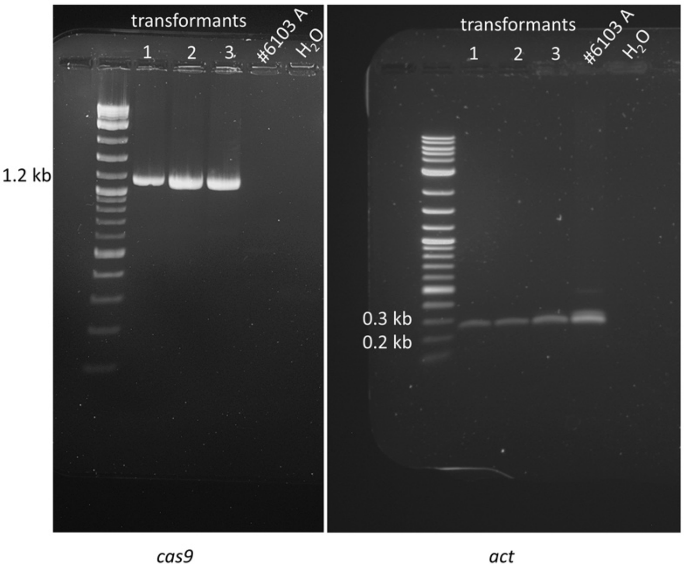

b

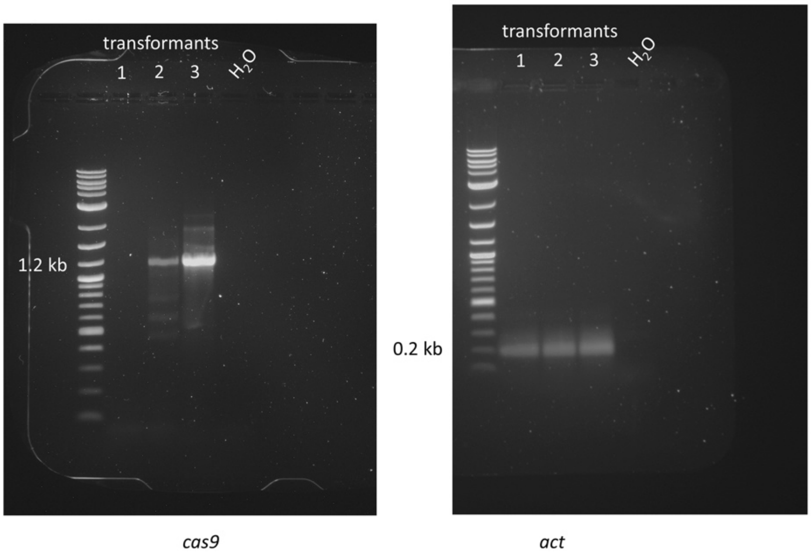

Supplementary Figure 1. Full-length gels of DNA-gels shown in Figure 1c and d. Gels for PCR (a) and RT-PCR (b)

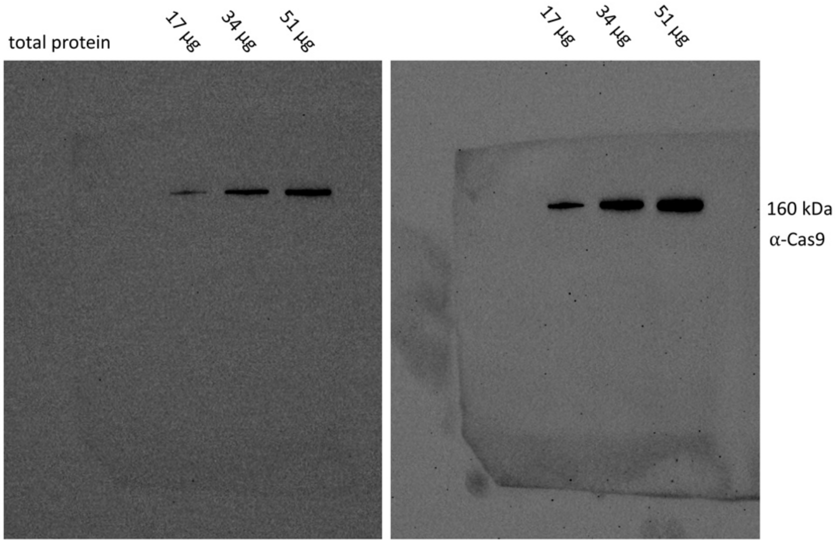

Supplementary Figure 2. Full-length blot of western blot shown in Figure 1e. Shown are two different exposures to make the membrane edges visible. The right side of the membrane was cut before antibody incubation, since the rest of the membrane belonged to another experiment.

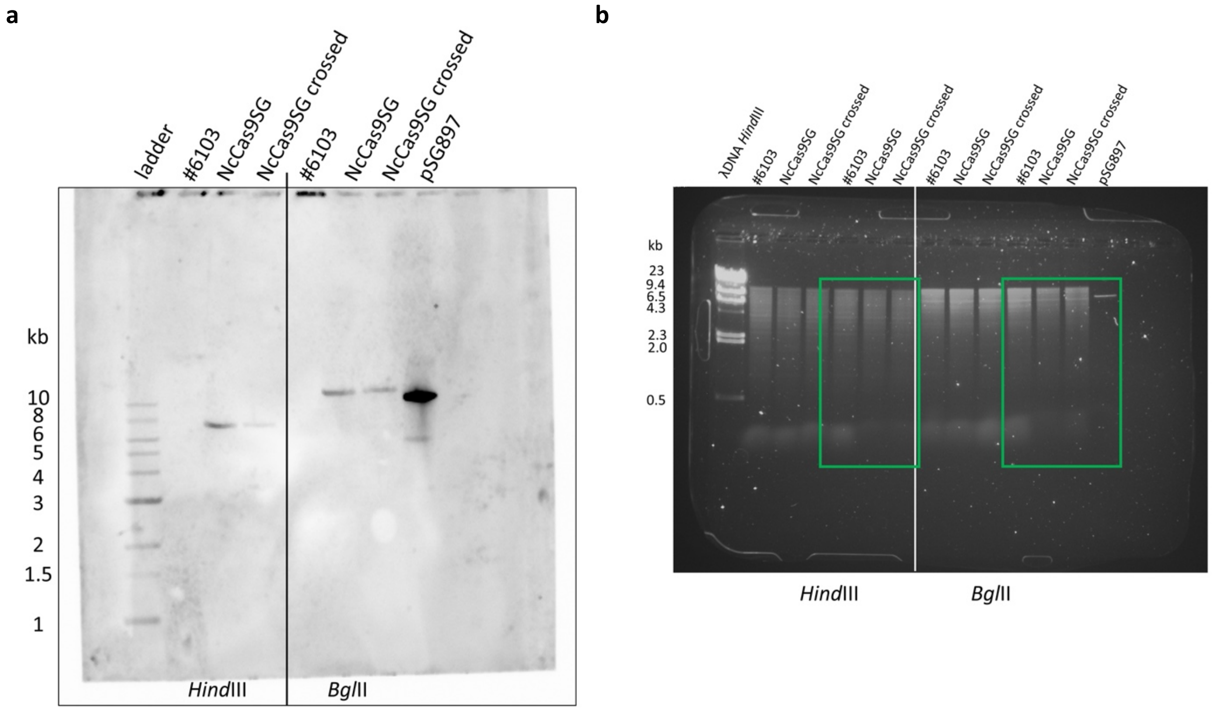

Supplementary Figure 3. Southern blot to confirm the *cas9* integration into the genome. Genomic DNA from #6103 the Cas9 transformant NcCas9SG used for all experiments and NcCas9SG crossed with #9716 were hydrolyzed with *Hind*III and *Bgl*II, respectively. A *cas9* probe targeting the transformation cassette was DIG-labeled and hybridized to the membrane. The bound probe was detected via chemiluminescence (**a**). Expected signals seen for #6103: none (hydrolyzed with *Hind*III and *Bgl*II). Expected signals seen for NcCas9SG and crossed strains: 7,355 bp (hydrolyzed with *Hind*III) and 11,399 bp

(hydrolyzed with *Bgl*II). Gel electrophoresis as a control for the hydrolyzation of the DNA used for the southern blot (green rectangle) (**b**).

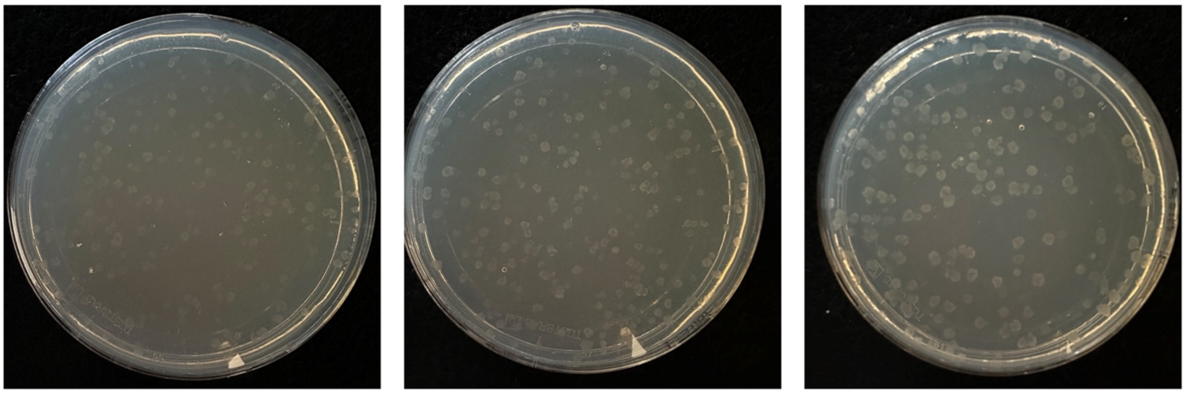

1 µg gRNA
223 colonies/plate ± 24

2 µg gRNA
246.4 colonies/plate ± 2.1

3 µg gRNA
209 colonies/plate ± 4.2

Supplementary Figure 4. *N. crassa* colonies on CsA containing VMM+SGF plates after transformation with different gRNA-c2 amounts
